# Integrating multi-informant reports of youth mental health: A construct validation test of Kraemer and colleagues’ (2003) Satellite Model

**DOI:** 10.3389/fpsyg.2022.911629

**Published:** 2022-07-28

**Authors:** Natalie R. Charamut, Sarah J. Racz, Mo Wang, Andres De Los Reyes

**Affiliations:** ^1^Comprehensive Assessment and Intervention Program, Department of Psychology, University of Maryland, College Park, College Park, MD, United States; ^2^Department of Management, University of Florida, Gainesville, FL, United States

**Keywords:** Converging Operations, Diverging Operations, informant discrepancies, Operations Triad Model, validity, Satellite Model

## Abstract

Accurately assessing youth mental health involves obtaining reports from multiple informants who typically display low levels of correspondence. This low correspondence may reflect *situational specificity*. That is, youth vary as to where they display mental health concerns and informants vary as to where and from what perspective they observe youth. Despite the frequent need to understand and interpret these *informant discrepancies*, no consensus guidelines exist for integrating informants’ reports. The path to building these guidelines starts with identifying factors that reliably predict the level and form of these informant discrepancies, and do so for theoretically and empirically relevant reasons. Yet, despite the knowledge of situational specificity, few approaches to integrating multi-informant data are well-equipped to account for these factors in measurement, and those that claim to be well-positioned to do so have undergone little empirical scrutiny. One promising approach was developed roughly 20 years ago by Kraemer and colleagues (2003). Their Satellite Model leverages principal components analysis (PCA) and strategic selection of informants to instantiate situational specificity in measurement, namely components reflecting variance attributable to the *context* in which informants observe behavior (e.g., home/non-home), the *perspective* from which they observe behavior (e.g., self/other), and behavior that manifests across contexts and perspectives (i.e., *trait*). The current study represents the first *construct validation* test of the Satellite Model. A mixed-clinical/community sample of 134 adolescents and their parents completed six parallel surveys of adolescent mental health. Adolescents also participated in a series of simulated social interactions with research personnel trained to act as same-age, unfamiliar peers. A third informant (*unfamiliar untrained observer*) viewed these interactions and completed the same surveys as parents and adolescents. We applied the Satellite Model to each set of surveys and observed high internal consistency estimates for each of the six-item *trait* (α = 0.90), *context* (α = 0.84), and *perspective* (α = 0.83) components. Scores reflecting the *trait*, *context*, and *perspective* components displayed distinct patterns of relations to a battery of criterion variables that varied in the context, perspective, and source of measurement. The Satellite Model instantiates situational specificity in measurement and facilitates unifying conceptual and measurement models of youth mental health.

## Introduction

Like a journalist writing a news story, assessing child and adolescent (i.e., youth) mental health involves attaining a holistic view of the youth undergoing evaluation. Thus, best practices in youth mental health assessment involve soliciting reports from multiple informants (Hunsley and Mash, 2007; De Los Reyes, 2011, 2013). Several decades ago, Achenbach et al. (1987) advanced the notion of *situational specificity* to characterize two key components of assessing youth mental health: (a) youth vary as to where they display mental health concerns and (b) the informants from whom assessors solicit reports (e.g., parents, teachers, youth, and their peers) vary in the contexts and perspectives from which they observe the youth. Consistent with this notion, informants’ reports of the same youth’s mental health tend to display relatively low levels of correspondence (i.e., mean *r* = 0.28; De Los Reyes et al., 2015). Evidence of these *informant discrepancies* traces back to the 1950s (e.g., Lapouse and Monk, 1958), manifests in assessments conducted globally (De Los Reyes et al., 2019a), and currently represents a literature that totals over 400 published studies (De Los Reyes and Makol, 2022).

Despite the frequent need to understand and interpret informant discrepancies, a persistent problem in measurement of youth mental health is that no consensus guidelines exist for integrating informants’ reports (Beidas et al., 2015). Without clarity in these areas, researchers and clinicians lack guidance on how to leverage multiple informants’ reports to make clinical decisions (Marsh et al., 2018; De Los Reyes et al., 2019b). *The central thesis of this paper is that the path to building these guidelines starts with identifying factors that reliably predict the level and form of these informant discrepancies, and do so for theoretically and empirically relevant reasons.* Yet, despite the knowledge of situational specificity, few approaches to integrating multi-informant data are well-equipped to account for these factors in measurement, and those that claim to be well-positioned to do so have undergone little empirical scrutiny.

In this paper, we address four aims. First, we review measurement and conceptual models for understanding informant discrepancies and integrating multi-informant data. Second, we use this review as a backdrop for considering an approach to integrating multi-informant data based on the notion of situational specificity (Kraemer et al., 2003). This approach holds promise, but has undergone surprisingly little validation testing. Third, we report findings of the first construct validation test of this integrative approach. Fourth, we describe the research, theoretical, and clinical implications of our construct validation study, and highlight directions for future work.

A key barrier to developing consensus guidelines lies with the most widely used approaches to integrating multi-informant data. As we have articulated elsewhere (for a recent review, see De Los Reyes et al., 2022a), the last 15 years of research very clearly point to a reality of multi-informant assessments conducted in youth mental health: The informant discrepancies produced by this approach often contain information relevant to understanding youth mental health (i.e., *domain-relevant information*). Yet, with few exceptions, available approaches do not account for this reality of multi-informant assessments.

In fact, the most commonly leveraged approaches to integrating multi-informant data stem from conceptual models (i.e., Converging Operations and the Multi-Trait Multi-Method Matrix [MTMM]; Garner et al., 1956; Campbell and Fiske, 1959) that take an extreme view of informant discrepancies, namely that they carry no value. Given the historically wide use of these models, in the past informant discrepancies were thought to largely reflect *measurement confounds* such as random error and rater biases (De Los Reyes, 2011; Dirks et al., 2012). Consider examples of the most commonly implemented integrative approaches. For instance, the composite score approach involves computing the sum or average of the informants’ scores (see Martel et al., 2021). Its key underlying assumption is that greater agreement among informants’ scores signals greater severity of the mental health concerns about which informants provide reports (e.g., De Los Reyes et al., 2016). Conversely, variance unique to any one source (i.e., as reflected by informant discrepancies) reflects error, and thus a core assumption underlying use of composite scoring is that estimates of psychological phenomena should emphasize shared or *common variance* among reports (see Edgeworth, 1888; Borsboom, 2005). This same rationale underlies applications of structural equation modeling (SEM) to integrating or modeling multi-informant data (see also Eid et al., 2008; Watts et al., 2021), combinational algorithms used to integrate data collected within diagnostic interviews (i.e., AND/OR rules; see Offord et al., 1996; Rubio-Stipec et al., 2003; Youngstrom et al., 2003; Valo and Tannock, 2010), and recent applications of measurement invariance techniques to detect informant discrepancies (e.g., Russell et al., 2016; Olino et al., 2018; Murray et al., 2021; Florean et al., 2022).

To be clear, there is nothing inherently wrong about these integrative approaches. Indeed, all statistical approaches have assumptions underlying their use; the key is understanding whether these usage assumptions “fit” the data conditions to which they will be applied. In this respect, if the most widely used approaches to integrating multi-informant data emphasize common variance, then they are optimized for application to data conditions in which estimates from informants’ reports agree to such an extent that they point to the same finding or conclusion in a study (i.e., Converging Operations). Consistent with this notion, when one uses approaches like composite scoring, SEM models that focus on estimating common variance, combinational algorithms, and measurement invariance techniques, one essentially adheres to key assumptions underlying Converging Operations (i.e., unique variance = measurement confounds). Yet, might assumptions underlying use of these integrative approaches be violated if valid data lies not only with instances in which estimates from informants’ reports agree, but also when they disagree?

The dominant procedures for integrating multi-informant data treat *all* informant discrepancies as measurement confounds, which begs the question: *Are all informant discrepancies created equally?* The Operations Triad Model (OTM; De Los Reyes et al., 2013) helps us build an evidence base to address this question. The OTM posits that the answer to this question is “no” and as such, provides users with a means for distinguishing at least two forms of informant discrepancies. Within the OTM, *Diverging Operations* denotes scenarios in which informant discrepancies reveal a facet of domain-relevant information that, by definition, cannot be considered a measurement confound. An example of such a scenario might be if parent and teacher reports of a youth’s hyperactivity disagree because the youth being assessed behaves differently at home and school, and the informants vary in where they observe the youth (i.e., situational specificity). Conversely, *Compensating Operations* denotes scenarios consistent with the integrative approaches described previously, namely that measurement confounds explain the discrepancies between informants’ reports. Examples might include different psychometric properties between informants’ reports (e.g., differences in internal consistency or score validity) or the presence of rater biases in one informant’s report to a greater degree than in the other informant’s report.

Taken together, the OTM delineates concepts (i.e., Converging, Diverging, and Compensating Operations) that researchers can leverage to pose hypotheses about what patterns of agreement and discrepancy between informants’ reports of youth mental health might reflect. Further, researchers have leveraged the OTM’s concepts to guide controlled tests of these hypotheses. For instance, consistent with the notion of situational specificity, when a parent reports elevated disruptive behavior in their child and the teacher does not, that child is highly likely to display disruptive behavior within parent-child interactions but not interactions between children and non-parental adults (De Los Reyes et al., 2009). In this case, the domain-relevant information revealed by the discrepancies (i.e., contextual variations in disruptive behavior) would be lost if the reports were integrated with a method that emphasizes common variance (e.g., composite scoring or SEM). In fact, a number of investigations point to informant discrepancies containing data that predict such domain-relevant criteria as pulmonary functioning (Al Ghriwati et al., 2018), treatment outcomes (Humphreys et al., 2017; Becker-Haimes et al., 2018; Makol et al., 2019; Zilcha-Mano et al., 2021), suicidal ideation (Augenstein et al., 2022), substance use risk (Lippold et al., 2014), and psychosocial impairments (De Los Reyes et al., 2022b). In sum, OTM-informed studies overwhelmingly indicate that at least some informant discrepancies reflect domain-relevant information.

The findings of OTM-informed studies beg another question: *What are the consequences of applying integrative approaches that only emphasize common variance to data conditions where domain-relevant variance resides in both common and unique variance?* When applied to data conditions that violate the assumption that only common variance matters—that unique variance cannot be domain-relevant—it is logical to hypothesize that using integrative approaches that carry the “only common variance matters” assumption has the consequence of depressing measurement validity (see also De Los Reyes et al., 2022c). Recent work supports such a hypothesis. For instance, integrative approaches that emphasize both common variance and domain-relevant unique variance outperform composite scores in terms of magnitudes of relations to criterion variables (De Los Reyes et al., 2022b) and direct tests of incremental validity (Makol et al., 2020). Further, the most commonly used MTMM-informed structural models cannot distinguish between informant discrepancies that reflect measurement confounds from those that reflect domain-relevant information (Watts et al., 2021). This work points to the need for guidance on approaches to integrating multi-informant data in youth mental health.

Operations Triad Model-informed research supports the development of guidelines for integrating multi-informant assessments of youth mental health. In particular, it appears that for many mental health domains, integrative approaches ought to optimize use of both common variance (i.e., Converging Operations) *and* domain-relevant unique variance (i.e., Diverging Operations), while minimizing the impact of measurement confounds (i.e., Compensating Operations). This notion begs yet another question: *Which integrative approaches account for both common variance and domain-relevant unique variance?* Roughly 20 years ago, Kraemer et al. (2003) proposed the “Satellite Model,” an approach to integrating multi-informant data that addresses the question of optimizing both common variance and domain-relevant unique variance. The authors explain their approach using methods underlying global positioning systems (GPS). Within a GPS, each satellite provides vital information when locating a target in space (e.g., building or a person), but only insofar as their location in space optimizes use of the information they provide. That is, one cannot obtain accurate location data using one well-placed satellite, or even multiple satellites placed at the same set of latitudes and longitudes. Rather, accurate location data comes from *triangulation*, such that each satellite’s position varies from the others based on predictable coordinates. For example, satellites 1 and 2 might be placed at the same latitude but disparate longitudes, whereas satellite 3 might be placed at a disparate latitude relative to 1 and 2, and at a longitude that “sits in between” the other satellites.

Kraemer et al. (2003) use satellite placement to explain a key idea: When normatively observing the presence of discrepant information, making sense of patterns of information necessitates *forcing* discrepancies to occur, using factors that reliably predict the discrepancies, and are based on domain-relevant aspects of the phenomena undergoing evaluation. When applied to multi-informant data, the integrative approach involves detecting factors analogous to the latitudes and longitudes used in satellite placement in GPS. In order to implement this approach, Kraemer and colleagues applied principal components analysis (PCA) to synthesize multi-informant data into orthogonal, domain-relevant factors that predictably result in informant discrepancies. Supported by several decades of research on moderators of correspondence between reports from multiple informants (Achenbach et al., 1987; De Los Reyes et al., 2015), the “latitudes and longitudes” in the Satellite Model consist of (a) the *context* in which an informant observes the youth undergoing evaluation and (b) the *perspective* (e.g., self vs. other) through which an informant observes the youth. Users of this approach select informants who vary in their contexts and perspectives, thus allowing for a third component (i.e., *trait*) to reflect *common variance*, namely aspects of youth mental health that generalize across informants’ contexts and perspectives. As such, the Satellite Model optimizes use of both *common variance* (i.e., *trait*) and domain-relevant *unique variance* (i.e., *context* and *perspective*).

As a tool for detecting patterns of common variance (i.e., *trait* component) and domain-relevant unique variance (i.e., *context* and *perspective* components), the Satellite Model optimizes use of both common variance and domain-relevant unique variance among informants’ reports to predict domain-relevant outcomes. Yet, this approach has rarely been subjected to validation testing, and thus two questions warrant consideration. First, to what degree does the approach reveal patterns of common variance and domain-relevant unique variance that manifest across mental health domains (e.g., depressive symptoms, anxiety, and psychosocial impairments)? The meta-analytic work on informant discrepancies points to these discrepancies manifesting regardless of domain (Achenbach et al., 1987; De Los Reyes et al., 2015). If so, then perhaps the path to increasing precision in estimating the “latitudes and longitudes” underlying this approach may be facilitated by applying the Satellite Model to a *battery* of multi-informant surveys. A battery of surveys would allow us to create multi-item scales of the *trait*, *context*, and *perspective* components derived from the Satellite Model, and to test their precision using well-established procedures (e.g., estimates of internal consistency; Nunnally and Bernstein, 1994).

Second, even if we discover that we can develop *precise* multi-item estimates of the *trait*, *context*, and *perspective* components derived from the Satellite Model, is it *accurate* to interpret these estimates as reflecting variations in informants’ contexts and perspectives? Indeed, this is a common issue in psychometrics and factor analysis. That is, using procedures like PCA to identify the factor structure of items only marks the first step in understanding variations among scores and the constructs they reflect (Messick, 1995; Pett et al., 2003; DiStefano and Hess, 2005; Strauss and Smith, 2009). Yet, researchers using the Satellite Model have subjectively interpreted scores reflecting the *trait*, *context*, and *perspective* components as if they directly reflect these components, without the underlying *construct validation* tests to support these interpretations. Essentially, users of the Satellite Model have engaged in the *naming fallacy*: “Just because a factor is named does not mean that the hypothetical construct is understood or even correctly labeled” (Kline, 2016, p. 300). In fact, we know of no previous study that has leveraged construct validation strategies to understand scores taken from the Satellite Model in relation to external criterion variables.

To address the two aforementioned gaps in the literature, we conducted a construct validation test of the Satellite Model with a battery of multi-informant assessments of adolescent social anxiety, consistent with recent work (Makol et al., 2020). Evidence-based assessments of internalizing concerns like social anxiety typically involve collecting reports from both parents and adolescents (Hunsley and Mash, 2007). In fact, evaluations of adolescent social anxiety often rely *exclusively* on parent and adolescent reports (De Los Reyes and Makol, 2019; Cannon et al., 2020). Parents and adolescents often disagree in their reports of adolescent social anxiety, and historically these discrepancies have been interpreted as the adolescent underreporting or downplaying their social anxiety symptoms, perhaps due to a social desirability bias (De Los Reyes et al., 2012a,^2015^). Yet, more parsimonious explanations might account for these discrepancies. For example, adolescent social anxiety often manifests in peer interactions, which makes this context an important part of assessment and treatment (Hofmann et al., 1999; Glenn et al., 2019). Importantly, relative to earlier developmental periods, parents have reduced opportunities to observe their adolescent outside of the home, particularly in terms of peer interactions (Smetana, 2008). Indeed, recent work finds that parent reports of adolescent social anxiety often fail to predict adolescents’ self-reported reactions to peer interactions (Deros et al., 2018). In this sense, parents’ lack of observation of their adolescent’s interactions with peers likely contributes to the discrepancies between parent and adolescent reports. When viewed through the lens of the Satellite Model, these findings indicate that, although parent and adolescent reports provide some “coverage” of adolescent social anxiety, effective triangulation requires an additional “satellite”: A third informant who simulates how a peer might observe the adolescent in a non-home context.

In an effort to collect survey data from this non-home informant, we recently developed the Unfamiliar Peer Paradigm (Cannon et al., 2020), a counterbalanced set of social interaction tasks designed to simulate adolescents’ social interactions with same-age, unfamiliar peers. Within this paradigm, adolescents interact with trained research personnel who display a youthful appearance consistent with the adolescent’s age (i.e., *peer confederates*). Adolescents interact with these peer confederates over a 20-min period. Following this period, unfamiliar untrained observers (UUOs) review video recordings of these interactions, and make reports using parallel versions of the measures completed by parents and adolescents. In essence, these UUOs comprise the third satellite involved in implementing the Satellite Model. In fact, recent work finds that UUOs’ reports of adolescents’ covert avoidance behaviors (i.e., safety behaviors) relate to both adolescents’ self-reports of these behaviors and adolescents’ social skills as displayed within the Unfamiliar Peer Paradigm (Rezeppa et al., 2021).

Building off of recent work (Makol et al., 2020; Rezeppa et al., 2021), the current study provides the first construct validation test of the Satellite Model for integrating scores taken from a battery of three informants’ reports (i.e., parents, adolescents, and UUOs). We leveraged a battery of six survey measures of adolescent mental health, and ran six independent PCAs in line with the Satellite Model. On these six sets of survey measures, adolescents, parents, and UUOs made reports about domains relevant to understanding adolescent social anxiety. Specifically, when adolescents experience social anxiety, they often also experience concerns with other internalizing domains, such as depression (see Epkins and Heckler, 2011). Further, adolescents who experience social anxiety often also experience psychosocial impairments stemming from their anxiety, along with several anxiety-relevant processes including fears of negative evaluation, and as mentioned previously, avoidance behaviors (see also Karp et al., 2018; Qasmieh et al., 2018; De Los Reyes and Makol, 2019; De Los Reyes et al., 2019c). Capitalizing on these common elements of adolescent social anxiety may optimize the precision of components assessed using the Satellite Model. That is, prior validation tests of the Satellite Model estimated components (i.e., *trait*, *context*, and *perspective*) using only a single set of multi-informant surveys (i.e., all reports about one domain; see Makol et al., 2020). In essence, this approach results in single-item measures of the component scores derived from the PCA. Importantly, a long line of research indicates that single-item measures display relatively weak psychometric properties, compared to multi-item measures (see Nunnally and Bernstein, 1994).

What if applications of the Satellite Model capitalized on a key observation made in informant discrepancies research? Specifically, within any two informants’ reports of the same youth, the *structure* of the discrepancies operates quite similarly across domains. That is, when examining informant discrepancies across domains rated within a given set of informants (i.e., the parent, teacher, and youth reports of a youth client’s mental health), the patterns of reports (e.g., parent > teacher; youth < parent) tend to generalize, even across distinct domains (e.g., internalizing vs. externalizing; De Los Reyes et al., 2016; Lerner et al., 2017; Makol et al., 2019, 2021). In these respects, we expect *trait*, *context*, and *perspective* scores from multi-informant assessments to display high levels of internal consistency across rated domains. We also expect that aggregating multi-domain *trait*, *context*, and *perspective* scores will optimize measurement precision.

A construct validation test of the Satellite Model also involves selecting a battery of *criterion variables* to test the domain-relevance of scores reflecting the model’s components. To return to the GPS analogy, if we treat informants’ reports as satellites that are strategically positioned in disparate points in space, then we must also think of the criterion variables in this way. The criterion variables must also display “satellite positions” that systematically vary. They ought to vary both from each other and in line with the *trait*, *context*, and *perspective* components used to synthesize the adolescent, parent, and UUO reports. In principle, this approach involves interpreting relations between the *trait*, *context*, and *perspective* components and criterion variables, based on the contexts and perspectives used to create the criterion variables. In practice, this approach involves leveraging criterion variables that tap multiple social contexts, perspectives, and measurement modalities. These criterion variables must also be domain-relevant or pertinent to understanding adolescent social anxiety.

Consequently, our construct validation test involved selecting three sets of domains and corresponding information sources that traversed home and non-home contexts, as well as the perspective of the rater. First, we leveraged a set of trained independent raters who observed adolescents within the Unfamiliar Peer Paradigm and rated their anxiety and social skills during the paradigm. In this way, we relied on a rater whose *perspective* was “neutral” or independent from that of the informants used to estimate the Satellite Model components (i.e., adolescents, parents, and UUOs). Further, the trained observers made ratings of adolescents’ behavior as it manifests in a non-home context. Second, family conflict is both ubiquitous to the adolescent period (see Smetana and Gaines, 1999; Adams and Laursen, 2001), and commonly manifests among adolescents who experience internalizing concerns (for a review, see Epkins and Heckler, 2011). Thus, we relied on parents’ reports of adolescent-parent conflict to collect information about a home-based psychosocial domain, rated from an observer perspective. Third, adolescents who experience social anxiety often also experience relatively high resting arousal (e.g., Monk et al., 2001; Thomas et al., 2012a). By construction, resting arousal is both an internal experience and occurs in a “neutral” setting, absent the environmental stimuli that typify adolescents’ daily home and non-home contexts. Thus, we relied on adolescents’ self-reports of resting arousal to collect information about a domain that is untethered from social context, and rated from a self-perspective. Collectively, this battery of criterion variables allowed us to test scores designed to reflect each of the Satellite Model’s *trait*, *context*, and *perspective* components.

We applied the Satellite Model to social anxiety assessments in a mixed-clinical/community sample of adolescents. Specifically, we extended the findings of Makol et al. (2020), who tested the approach using a single set of multi-informant reports of adolescent social anxiety. We addressed four aims. First, we examined levels of correspondence among parent, adolescent, and UUO reports. As with prior work (Achenbach et al., 1987; De Los Reyes et al., 2015), we expected to observe low-to-moderate levels of correspondence across domains. Second, we scored parent-adolescent-UUO triads derived from six sets of multi-informant assessments using the Satellite Model. Third, we tested the internal consistencies of the aggregated or “total scores” for both the common variance component (i.e., total *trait* score) as well as the unique variance components (i.e., total *context* and *perspective* scores). We expected these total scores to display acceptable levels of internal consistency.

Fourth, we tested the bivariate and unique relations of the aggregated *trait*, *context*, and *perspective* component scores and a series of criterion variables that, collectively, comprised a construct validation test of these scores. In prior work (Makol et al., 2020), a *trait* score based solely on three reports of adolescent social anxiety demonstrated criterion-related validity when predicting observed anxiety. Theoretically, the Satellite Model optimizes both common variance (i.e., *trait* score) and domain-relevant unique variance in prediction, with two components that capture distinct facets of this unique variance (i.e., *context* vs. *perspective*). As such, we expected the Satellite Model scores to differentially relate to our battery of criterion variables, depending on the context and perspective the criterion variable was designed to reflect.

Specifically, we expected independent observers’ ratings of adolescent social anxiety and social skills to uniquely relate to scores reflecting the *trait* and *context* components. The basis of this prediction lies in two aspects of our independent observers’ ratings. First, prior work indicates that all three informants’ reports (i.e., parents, adolescents, and UUOs) each relate to independent observers’ ratings (see Glenn et al., 2019; Makol et al., 2020; Rezeppa et al., 2021). In this respect, relations between these reports and ratings of observed behavior generalize across informants’ contexts and perspectives (i.e., *trait* score). However, the Unfamiliar Peer Paradigm from which independent observers base their ratings nonetheless is based exclusively on behaviors displayed in non-home contexts (i.e., unfamiliar peer interactions). Thus, we would also expect the *context* component to uniquely relate to observers’ ratings. Conversely, we would not expect a link between the *perspective* component and independent observers’ ratings, given that independent observers’ perspectives, by construction, are not represented among the perspectives of the three informants.

In contrast to our first set of predictions regarding independent observers’ ratings, we expected two different patterns of relations between the *trait*, *context*, and *perspective* scores and criterion variables designed to capture aspects of the *context* and *perspective* components. Specifically, we expected parents’ reports of adolescent-parent conflict to uniquely relate to scores reflecting the *context* and *perspective* components, because (a) like behaviors displayed in the Unfamiliar Peer Paradigm, adolescent-parent conflict manifests in a specific context (i.e., the home) and (b) we relied on parent reports (i.e., a unique, observer perspective) to provide reports about conflict. Further, we expected adolescents’ self-reported resting arousal to uniquely relate to scores reflecting the *perspective* component. This is because (a) resting arousal reflects a process that is untethered to contextual factors (i.e., no relation to the *context* component) and (b) like parent-reported conflict, we relied on a single unique perspective (i.e., adolescent self-report) to assess resting arousal.

## Materials and methods

Participants were 134 adolescents aged 14–15 years old (*M* = 14.5 years; *SD* = 0.5) and their parents who were recruited as part of a larger study (e.g., Deros et al., 2018; Glenn et al., 2019; Cannon et al., 2020). In order to participate in the study, parent-child dyads had to meet the following inclusion criteria: (a) speak English, (b) have a 14–15-year-old adolescent living at home, and (c) understand the consent and assent process. Within this sample, 89 adolescents identified as female and 45 identified as male. Parents identified their adolescent’s race/ethnicity (African American or Black: 53%; White, Caucasian American, or European: 34%; Asian American or Asian: 5%; Hispanic or Latino/a (Spanish): 10%; American Indian: 0.7%; or “Other”: 7%). The parents could select multiple racial/ethnic backgrounds, leading to these rates totaling over 100%. Parents reported weekly household income in increments of $100 (e.g., $101–$200 per week) with the following breakdown: 26% of parents earned $500 or less per week, 22% earned between $501 and $900 per week, and 51% earned more than $901 weekly. Parents reported marital status with 50% currently married, 21% never married, 16% divorced, 8% separated, and 0.7% widowed. Parents also reported their highest level of education, with 3% less than high school or equivalent, 14% a high school diploma or equivalent (i.e., GED), 17% some college, 10% an associate’s or vocational degree, 19% a bachelor’s degree, 23% a master’s degree, and 13% an advanced degree.

The Institutional Review Board of the large mid-Atlantic university where the study was conducted approved the procedures of the study prior to administration. Participants were recruited from Maryland, Washington D.C., and Northern Virginia through public advertisements. We recruited participants through a variety of methods including advertisements online (e.g., Craigslist, the laboratory website, Facebook, and Google Ads), on public transportation (e.g., busses, Metro rail, and Metro stations), and in local spaces (e.g., flyers posted in the community including bulletin boards and community Listservs, cards handed out during campus events). Recruitment also took place at the offices of local health professionals (i.e., doctor’s offices, clinics, and hospitals) who serve the targeted population.

Two different advertisements were used to recruit participants, with one depicting a no-cost screening clinical assessment for evaluation of adolescent social anxiety (i.e., clinic-referred adolescents; *n* = 45) and the other depicting a study assessing parent-child interactions (i.e., community control adolescents; *n* = 89). Both groups completed the same assessments and tasks described below during an in-person laboratory visit. Following the visit, parents in the clinic-referred group received feedback on their adolescent’s functioning and referrals for treatment, whereas those in the community control group did not receive feedback/referrals.

For this study, we used an analytic approach that pooled the two groups into one sample. By combining these two groups, we capitalized on key features of adolescent mental health concerns, namely that they dimensionally vary in the general population (i.e., fewer numbers of adolescents displaying concerns relative to those not displaying concerns, and scores ranging from relatively low concerns to relatively high concerns). This is an approach we have taken in multiple studies leveraging this same sample (e.g., Botkin et al., 2021; Okuno et al., 2021; Greenberg and De Los Reyes, 2022), including the study our current study seeks to extend (Makol et al., 2020). Further, this approach is consistent with both current initiatives focused on dimensional models of psychopathology (e.g., Insel et al., 2010), and prior work indicating enhanced reliability and validity for dimensional approaches to measuring and examining psychopathology, relative to discrete approaches (e.g., testing aims separately within subgroups; Markon et al., 2011). Importantly, prior work indicates this approach results in clinic-referred and community control groups that display comparable demographic characteristics, thus further justifying use of this approach (see Cannon et al., 2020; Makol et al., 2020). Demographic data for these groups are available upon request from the corresponding author.

Parents completed an initial phone screen with the laboratory staff to assess if they and their adolescent met our inclusion criteria. If they met the criteria, we scheduled them to complete assessments in the laboratory. For the in-person assessment, research personnel described the study and provided parental consent and adolescent assent forms to review and sign. Following consent/assent, adolescents and parents completed parallel sets of survey measures independently on computers in counterbalanced order using the Qualtrics survey platform. Additionally, adolescents completed three counterbalanced social interactions tasks with study personnel trained to interact with the adolescents as unfamiliar peer confederates (see Cannon et al., 2020). Upon completing the study tasks, families received a total of $100 in monetary compensation ($50 for the parent and $50 for the adolescent).

### Instruments

#### Unfamiliar peer paradigm

Adolescents interacted with unfamiliar peer confederates in the Unfamiliar Peer Paradigm (Cannon et al., 2020). The Unfamiliar Peer Paradigm is a series of counterbalanced social interaction tasks with trained, gender-matched research assistants designed to be reflective of interactions with same-age, unfamiliar peers. Peer confederates had no prior contact with the adolescent with whom they interacted, and we masked peer confederates to adolescents’ referral status and all other clinical information related to adolescent participants. The tasks included a series of structured, dyadic role-plays between adolescents and peer confederates (i.e., Simulated Social Interaction Test [SSIT]), an unstructured dyadic conversation between adolescents and peer confederates designed to simulate the first day of class (i.e., Unstructured Conversation Task [UCT]), and a public speaking task in which adolescents spoke about a series of predetermined social issues (i.e., Impromptu Speech Task [IST]). Each of these tasks have been described at length in prior published work (e.g., Deros et al., 2018; Glenn et al., 2019; Makol et al., 2020), and Cannon et al. (2020) provided an overview of the overall paradigm and empirical support for its use. Additionally, scripted procedures for the Unfamiliar Peer Paradigm’s tasks exist on the Open Science Framework Platform (De Los Reyes, 2020).

##### Unfamiliar untrained observers’ reports of adolescent mental health

Using archival videos of the adolescent’s participation in the Unfamiliar Peer Paradigm, we randomly assigned UUOs to view up to five recordings of the social interaction tasks. After viewing the recordings, UUOs made survey reports about each adolescent using the battery of six survey measures described below. Importantly, UUOs received no training on how to make these survey reports. In this respect, they received measure instructions akin to the parents and adolescents involved in the study. We masked UUOs to adolescents’ referral status and all other clinical information. In online Supplementary Material, we report the demographic characteristics of the UUOs who completed reports.

#### Satellite Model survey battery

Adolescents, parents, and UUOs completed survey measures across a battery of psychosocial domains. All informants completed the measures from their own perspective on reporting about the adolescent. That is, all survey measures included parallel item content for all informants, with only minor word modifications to fit their perspective (e.g., “I” for adolescent vs. “My child” for parent vs. “The participant” for UUO). Adolescents and parents completed survey measures immediately following completion of consent/assent forms and before administration of the Unfamiliar Peer Paradigm. UUOs completed these same measures based on observations of the adolescents’ behavior during video recordings of the Unfamiliar Peer Paradigm. Extensive descriptions of each of the measures in our battery (i.e., psychometric properties, example items, response options) can be found in prior work (see Rausch et al., 2017; Deros et al., 2018; Karp et al., 2018; Qasmieh et al., 2018; De Los Reyes et al., 2019c; Botkin et al., 2021; Rezeppa et al., 2021).

##### Social interaction anxiety scale

The Social Interaction Anxiety Scale (SIAS; Mattick and Clarke, 1998) is a 20-item measure of social anxiety displayed during direct social interaction. Informants made responses on a 5-point Likert scale ranging from “0” to “4” with higher scores indicating higher levels of social anxiety. Parents’ and adolescents’ SIAS reports display high levels of internal consistency (α > 0.90) and distinguish adolescents on referral status (Deros et al., 2018).

##### Social phobia scale

The Social Phobia Scale (SPS; Mattick and Clarke, 1998) is a 20-item scale measuring concerns related to social anxiety regarding everyday behaviors. Each item is rated on a Likert scale that ranges from “0” to “4” with higher scores indicating higher levels of social anxiety. Similar to the SIAS, parents’ and adolescents’ SPS reports display high levels of internal consistency (α > 0.90) and distinguish adolescents on referral status (Deros et al., 2018).

##### Brief fear of negative evaluation scale

The Brief Fear of Negative Evaluation Scale (BFNE; Leary, 1983) measures fears related to negative evaluation from other individuals. The BFNE is a 12-item measure rated on a 5-point Likert scale ranging from “1” to “5” with higher scores indicating higher evaluative fears. Parents’ and adolescents’ BFNE reports display high levels of internal consistency (α > 0.80) and distinguish adolescents on referral status (Karp et al., 2018; Szollos et al., 2019).

##### Subtle avoidance frequency examination

The Subtle Avoidance Frequency Examination (SAFE; Cuming et al., 2009) is a 32-item measure where each item describes a safety behavior that could be employed during a social interaction. Informants indicate the frequency the different safety behaviors on a 5-point scale ranging from “1” to “5” with higher scores indicating higher levels of safety behaviors. Informants’ reports of adolescents on the SAFE display high levels of internal consistency (α > 0.80) and distinguish adolescents’ on referral status (Thomas et al., 2012b; Qasmieh et al., 2018; Rezeppa et al., 2021).

##### Work and social adjustment scales for youth

The Work and Social Adjustment Scales for Youth (WSASY; De Los Reyes et al., 2019c) assesses adolescents’ psychosocial impairments. It contains 5 items assessing the adolescent’s behavior without mention of mental health concerns or status (e.g., *“Because of the ways I think, feel or behave, my ability to do well in school is impaired.”*). Severity of impairment is indicated using a Likert scale from “0” to “8” with higher scores indicating greater levels of impairment. Parents’ and adolescents’ WSASY reports display high levels of internal consistency (α > 0.80) and distinguish adolescents on the number of peer-related impairments (De Los Reyes et al., 2019c).

##### Beck depression inventory-II

The Beck Depression Inventory-II (BDI-II; Beck et al., 1996) is a widely used measure of depressive symptoms. The measure was originally designed for use with participants aged 13 years and older, and recent work supports its psychometric properties when administered to adolescents (e.g., Rausch et al., 2017; Qasmieh et al., 2018; Glenn et al., 2019). Respondents rate items describing depressive symptoms (e.g., sadness, loss of interest, feelings of guilt) on a 4-point scale with higher scores indicating greater depressive symptoms. As in prior work (e.g., Rausch et al., 2017; Deros et al., 2018), we excluded two items (9 and 21) which assess for suicidality and loss of interest in sex, as parents often decline to consent to their children reporting on these items due to their mature content. Despite excluding these items, sample internal consistency estimates remained high (Table 1).

**TABLE 1:** Means (M), standard deviations (SD), and internal consistency estimates (α) of survey measures used to estimate Satellite Model components.

Measure	*M*	*SD*	α
**Social Interaction Anxiety Scale**			
Adolescent Self-Report	28.04	16.14	0.93
Parent Report about Adolescent	27.04	16.54	0.95
Unfamiliar Untrained Observer	42.22	18.84	0.96
**Social Phobia Scale**			
Adolescent Self-Report	21.42	15.41	0.93
Parent Report about Adolescent	16.91	14.38	0.94
Unfamiliar Untrained Observer	30.88	18.03	0.96
**Brief Fear of Negative Evaluation**			
Adolescent Self-Report	34.80	9.18	0.87
Parent Report about Adolescent	34.40	9.69	0.90
Unfamiliar Untrained Observer	39.26	9.63	0.92
**Subtle Avoidance Frequency Examination**			
Adolescent Self-Report	66.19	20.24	0.93
Parent Report about Adolescent	64.74	17.43	0.92
Unfamiliar Untrained Observer	77.02	18.29	0.91
**Work and Social Adjustment Scale for Youth**			
Adolescent Self-Report	10.07	8.07	0.84
Parent Report about Adolescent	8.83	7.71	0.84
Unfamiliar Untrained Observer	10.90	7.38	0.85
**Beck Depression Inventory-II**			
Adolescent Self-Report (Raw Score)	13.04	10.72	0.92
Adolescent Self-Report (Square Root Transformed Score)	3.31	1.45	−
Parent Report about Adolescent (Raw Score)	6.81	7.95	0.90
Parent Report about Adolescent (Square Root Transformed Score)	2.08	1.58	−
Unfamiliar Untrained Observer (Raw Score)	11.54	10.10	0.94
Unfamiliar Untrained Observer (Square Root Transformed Score)	3.00	1.60	−

All study aims addressed using Beck Depression Inventory-II scores were based on the square root transformed scores.

#### Criterion measures for construct validation

##### Independent observers’ ratings of adolescents’ social anxiety and social skills

We leveraged behavioral ratings from trained independent observers to assess adolescents’ social anxiety and social skills within the Unfamiliar Peer Paradigm. By construction, these trained independent observers differed from each of the survey informants in two key ways. First, unlike parents, our trained independent observers made ratings that were based specifically on behaviors displayed in a non-home context. Second, although our trained independent observers displayed some overlap in their context of observation with adolescents, and complete contextual overlap with UUOs, they made ratings with the benefit of training. In this respect, the perspective from which our trained independent observers made ratings differed from all three informants. Thus, these characteristics of trained independent observers comprised one facet of our larger construct validation test of the Satellite Model.

The trained independent observers consisted of undergraduate and post-baccalaureate research assistants who did not participate in any of the social interaction tasks as a peer confederate and did not complete survey reports as a UUO. As with UUOs, we masked independent observers to adolescents’ referral status and all other clinical information. We provided extensive information on coder training and characteristics in online Supplementary Material, as well as in prior work (Glenn et al., 2019; Cannon et al., 2020; Botkin et al., 2021).

Independent observers made global ratings of each adolescent’s social anxiety and social skills using an extensively validated behavioral coding scheme (e.g., Glenn et al., 2019). For each domain, independent observers based their ratings on observations of the SSIT (five ratings), UCT (one rating), and IST (one rating). Independent observers made social anxiety ratings on a 5-point scale ranging from 1 (Animated) to 5 (Severe anxiety), where higher scores indicated greater social anxiety. Further, independent observers made social skill ratings on a 5-point scale ranging from 1 (*Not effective at all*) to 5 (*Very effective*), where higher scores indicated greater social skills. For each adolescent, a pair of coders rated their social anxiety and social skills, with ratings displaying *ICC*s of 0.75 and 0.81, respectively (“excellent” range per Cicchetti, 1994).

For each adolescent, we computed composite scores for each of the seven task ratings (5 SSIT, 1 UCT, 1 IST) by taking an average of the pair of the independent observers’ ratings. Although we computed composite scores for all 134 adolescents, some were missing data on one task rating (e.g., one of the five SSIT ratings), whereas three adolescents declined to give a speech for the IST. Consistent with prior work (Makol et al., 2020) and to reduce Type 1 Error, we created composite mean scores for all seven social anxiety ratings (*M* = 3.09, *SD* = 0.82) and seven social skills ratings (*M* = 3.51, *SD* = 0.89). For adolescents for whom we were missing data on these tasks, we computed their composite scores based on the six ratings we had available for them. In terms of psychometric support, independent observers’ ratings relate to well-established survey measures of adolescent social anxiety and related processes (e.g., safety behaviors, fears of evaluation, psychosocial impairments) and distinguish adolescents on referral status (e.g., Glenn et al., 2019; Cannon et al., 2020; Botkin et al., 2021; Rezeppa et al., 2021).

##### Parent-reported adolescent-parent conflict

To assess parent-adolescent conflict, parents completed the Issues Checklist (IC; Prinz et al., 1979). For the purposes of our study, parent-reported conflict data using the IC allowed us to measure a psychosocial domain that (a) is based specifically in the home context, (b) reflects external events (i.e., conflict), and (c) uses the lived experience of an informant whose report comes from an observer perspective. Thus, these characteristics of parent reports on the IC comprised the second facet of our construct validation test of the Satellite Model. On the IC, parents report on topics of disagreement within the past 4 weeks. We modified the IC for the purposes of time (i.e., reduce participant burden) and to assess ranges of conflict related to topics about which parents and adolescents typically encounter at home (e.g., chores, homework, and friends), as consistent with prior work (e.g., Smetana and Gaines, 1999; Adams and Laursen, 2001; Treutler and Epkins, 2003; Ehrlich et al., 2011; De Los Reyes et al., 2012b). Specifically, our modified checklist included 16 of the 44 topics listed on the original IC. A list of the 16 IC topics we assessed is available from the corresponding author. We also modified the response format so that parents could rate conflict about each topic using a 5-point Likert-type scale ranging from 1 (we do not disagree) to 5 (we disagree much). Parents completed the checklist with regard to conflicts between themselves and the adolescent with whom they participated in the study, and vice versa for the adolescent. For this study, we calculated total scores by summing the scores across the 16 items, with possible total scores ranging from 16 to 80 (*M* = 33.66, *SD* = 12.01). The psychometric properties of the IC used in this study and evidence of its reliability and validity have previously been reported (Ehrlich et al., 2011; De Los Reyes et al., 2012b; Rausch et al., 2017).

##### Adolescent self-reported resting arousal

Adolescents reported self-perceived levels of internal arousal using the Self-Assessment Manikin (SAM; Lang, 1980). The SAM is a 5-level pictorial scale of affect ranging from 1 (close-eyed/relaxed image) to 5 (wide-eyed/nervous image). Adolescents completed a rating of their *resting* arousal at a baseline period before administration of the Unfamiliar Peer Paradigm (*M* = 1.55; *SD* = 0.62). In this way, we could collect an arousal rating based on a psychological process (resting state) that is (a) stripped of all contextual information, (b) based on an internal process and thus (c) based on the lived experiences of an informant whose report comes from a self-perspective. These characteristics of adolescent self-reports on the SAM comprised the third facet of our construct validation test of the Satellite Model.

### Data analyses

#### Preliminary analyses

We followed a multi-step plan for addressing our aims. First, each of our measures consisted of either multi-item surveys of unidimensional constructs or ratings of adolescent behavior for which we calculated composite scores (i.e., of two independent observers’ ratings for each adolescent). Thus, consistent with prior work using these measures (e.g., Thomas et al., 2012b; Deros et al., 2018; Qasmieh et al., 2018; Glenn et al., 2019), and to produce estimates to compare against prior work, we assessed the reliability of scores taken from these measures by calculating estimates of either internal consistency (Cronbach’s α for survey measures) or inter-rater reliability (*ICC*s for independent observers’ ratings). We interpreted these calculations relative to conventions for α (e.g., Nunnally and Bernstein, 1994) and *ICC*s (e.g., Cicchetti, 1994). We then computed means and standard deviations for all continuous measures, and calculated statistics for skewness and kurtosis to determine if our data met assumptions for our planned parametric analyses (i.e., skewness/kurtosis in range of ±2.0; Tabachnick and Fidell, 2001).

#### Cross-informant correspondence

Second, to estimate cross-informant correspondence on survey reports of adolescent mental health, we computed Pearson *r* correlations among adolescent, parent, and UUO reports on parallel measures (e.g., adolescent-parent, adolescent-UUO, and parent-UUO correlations of SIAS reports).

#### Scoring multi-informant assessment battery of adolescent mental health

Third, to prepare multi-informant assessments for the PCA of the Satellite Model components, we scored adolescent, parent, and UUO reports on parallel measures (e.g., the three reports collected on the SPS) using the approach described by Kraemer et al. (2003). As mentioned previously, we used a set of informants who collectively varied in their contexts and perspectives, with (a) informants observing from a home-based, observer perspective (parents); (b) informants observing from a non-home-based, observer perspective (UUOs); and (c) informants observing from a self-perspective based on a mix of home and non-home contexts. As such, we expected our PCA to include a *trait* score component in which all informants’ reports load strongly and in the same direction. We also expected our PCA to reveal a *context* score (i.e., informants from different contexts load in opposite directions) as well as a *perspective* score (i.e., self-reports load in the opposite direction of observer informants’ reports). Consistent with Kraemer et al. (2003) and recent work by Makol et al. (2020), we conducted six unrotated PCAs, one for each of the parallel measures in our multi-informant battery (i.e., SIAS, SPS, BFNE, SAFE, WSASY, and BDI-II). Each of these PCAs essentially consisted of three “items,” namely the adolescent, parent, and UUO reports on the same survey measure. In this respect, for each PCA, our subject-to-item ratio (i.e., 134/3 = 44.67:1) was well above the typical subject-to-item ratios deemed “large” within PCA modeling contexts (e.g., 20:1; see Osborne and Costello, 2004). Within these six unrotated PCAs, we set the number of components to be extracted to three. We examined principal component weights for each informant’s report to determine whether we identified the *trait*, *context*, and *perspective* scores described previously.

#### Internal consistency for the Satellite Model components and creation of total scores

Fourth, we computed α statistics for each six-item set of component scores (i.e., one α per component domain). In preparation for our construct validation test of the Satellite Model, we computed three total scores, one for each Satellite Model component (e.g., total summation of the six *Context* score items).

#### Relations between the Satellite Model components and criterion variables

Fifth, to test the criterion-related validity of scores taken from the Satellite Model, we computed a series of Pearson *r* correlations. The correlations estimated relations between the Satellite Model scores and criterion variables. For any one set of bivariate tests, if two or more components bore a relation to the criterion variable, we wanted to ensure that each component *uniquely* related to the criterion variable. Thus, we constructed linear regression models to estimate unique relations between Satellite Model scores and that criterion variable. In these models, we entered the Satellite Model scores in separate steps, whereby we entered the common variance estimate (i.e., *trait* score) in the first step. We entered any unique variance estimates (i.e., *context* and/or *perspective* scores) separately, in subsequent steps.

## Results

### Preliminary analyses

To determine if any of our study variables deviated significantly from normality (i.e., skewness and kurtosis), we conducted a descriptive analysis of adolescent, parent, and UUO responses to all surveys, as well as scores for all of our criterion variables. With one exception, the data met basic assumptions of parametric statistical tests (skewness/kurtosis in range of ± 2.0). Specifically, all three informants’ reports on the BDI-II displayed significant skewness and/or kurtosis. We addressed these concerns by applying a square root transformation to all BDI-II reports, which brought them all underneath the thresholds reported previously. All analyses reported below use these transformed scores. Table 1 displays the means and standard deviations for informants’ reports on all survey measures. Table 1 also displays α estimates for all informants’ survey reports, which all displayed acceptable levels (α > 0.08; Nunnally and Bernstein, 1994).

### Cross-informant correspondence

In Table 2, we report bivariate correlations among adolescent, parent, and UUO reports on the six survey measures. Supporting previous work, we observed low-to-moderate correlations among informants’ reports (Achenbach et al., 1987; De Los Reyes et al., 2015).

**TABLE 2:** Bivariate correlations among informants’ reports on parallel measures.

Measure	*Adolescent-Parent*	*Adolescent-Unfamiliar untrained observer*	*Parent-Unfamiliar untrained observer*
Social Interaction Anxiety Scale	0.39***	0.32***	0.24**
Social Phobia Scale	0.31***	0.31***	0.19*
Brief Fear of Negative Evaluation	0.32***	0.14	−0.07
Subtle Avoidance Frequency Examination	0.33***	0.22*	−0.04
Work and Social Adjustment Scale for Youth	0.27**	0.12	0.06
Beck Depression Inventory-II	0.34***	0.10	0.003

*p < 0.05; **p < 0.01; ***p < 0.001.

### Scoring multi-informant assessment battery of adolescent mental health

In Table 3, we report the eigenvalues and component loadings for the PCAs of the six sets of adolescent, parent, and UUO survey reports. Consistent with prior work (e.g., Kraemer et al., 2003; Makol et al., 2020), these PCA models each revealed loadings consistent with the *trait*, *context*, and *perspective* components as described previously. Specifically, all informants’ reports loaded positively onto the *trait* component, informants’ reports from different contexts (i.e., parent vs. UUO) loaded onto the *context* component in opposite directions, and adolescent self-reports loaded onto the *perspective* component in a direction opposite of the loadings observed from the two observer informants (i.e., parent and UUO).

**TABLE 3:** Eigenvalues and principal components analysis loadings for the 18-item Satellite Model.

Item	Eigenvalue	Adolescent	Parent	UUO
Social Interaction Anxiety Scale *Trait* Score	1.64	0.79	0.74	0.68
Social Phobia Scale *Trait* Score	1.54	0.78	0.68	0.68
Brief Fear of Negative Evaluation *Trait* Score	1.32	0.83	0.77	0.17
Subtle Avoidance Frequency Examination *Trait* Score	1.38	0.85	0.70	0.41
Work and Social Adjustment Scale for Youth *Trait* Score	1.32	0.77	0.73	0.44
Beck Depression Inventory-II *Trait* Score	1.35	0.82	0.79	0.23

Social Interaction Anxiety Scale *Context* Score	0.77	−0.15	−0.49	0.71
Social Phobia Scale *Context* Score	0.81	−0.003	−0.63	0.64
Brief Fear of Negative Evaluation *Context* Score	1.05	0.16	−0.38	0.94
Subtle Avoidance Frequency Examination *Context* Score	1.04	0.04	−0.56	0.85
Work and Social Adjustment Scale for Youth *Context* Score	0.95	−0.13	−0.39	0.89
Beck Depression Inventory-II *Context* Score	1.00	−0.01	−0.27	0.96

Social Interaction Anxiety Scale *Perspective* Score	0.59	−0.59	0.46	0.19
Social Phobia Scale *Perspective* Score	0.65	−0.63	0.36	0.35
Brief Fear of Negative Evaluation *Perspective* Score	0.62	−0.53	0.50	0.29
Subtle Avoidance Frequency Examination *Perspective* Score	0.58	−0.52	0.45	0.32
Work and Social Adjustment Scale for Youth *Perspective* Score	0.72	−0.61	0.56	0.15
Beck Depression Inventory-II *Perspective* Score	0.65	−0.57	0.55	0.15

UUO, Unfamiliar Untrained Observer.

### Internal consistency for the Satellite Model components and creation of total scores

We computed α estimates for the six-item *trait*, *context*, and *perspective* scales. The six-item *trait* scale displayed an α of 0.90 and a mean inter-item correlation of 0.61. The six-item *context* scale displayed an α of 0.84 and a mean inter-item correlation of 0.47. The six-item *perspective* scale displayed an α of 0.83 and a mean inter-item correlation of 0.44. Thus, each scale displayed high internal consistency (i.e., α > 0.80; see Nunnally and Bernstein, 1994), particularly for short, six-item scales (see also Youngstrom et al., 2019). Based on these findings, we computed total scores for each of the Satellite Model component scales by summing up each of the six-item scales for *trait* (*M* = 0; *SD* = 4.92; minimum value = −8.17; maximum value = 15.55), *context* (*M* = 0; *SD* = 4.50; minimum value = −13.11; maximum value = 11.16), and *perspective* (*M* = 0; *SD* = 4.40; minimum value = −9.33; maximum value = 10.34). All means for components derived from PCA are zero because of their standardization.

### Demonstrating the construct validity of scores reflecting the Satellite Model components

Using the *trait*, *context*, and *perspective* total scores described previously, we conducted our construct validation test of the Satellite Model. In Table 4, we report a summary of the findings and specify the direction of significant effects. Below, we describe each set of findings separately, by criterion variable, first in terms of the bivariate relations tested using correlations, and for those significant bivariate relations, we report findings from hierarchical multiple regressions testing for unique relations.

**TABLE 4:** Summary of findings for construct validation test of the Satellite Model.

		Criterion variables used for construct validation test (Source, context, and perspective of measurement)	
Satellite model component	*Social anxiety and social skills* (Trained observer, non-home context, neutral perspective)	*Adolescent-parent conflict* (Parent, home context, observer perspective)	*Adolescent resting arousal* (Adolescent, neutral context, self-perspective)
*Trait*	• Greater *trait* scores, greater anxiety and lower social skills *	×	• Greater *trait* scores, greater resting arousal *

*Context*	• Greater *context* scores (i.e., direction of more adolescent concerns rated by informant in the *non-home context*), greater anxiety and lower social skills *	• Lower *context* scores (i.e., direction of more adolescent concerns rated by informant in the *home context*), greater conflict *	×

*Perspective*	×	• Greater *perspective* scores (i.e., direction of more adolescent concerns rated by *observer-report informant*), greater conflict *	• Lower *perspective* scores (i.e., direction of more adolescent concerns rated by *self-report informant*), greater arousal *

*Statistically significant, unique effect; ×= null effect.

#### Construct validity of scores reflecting the trait and context components

##### Bivariate relations

Bivariate correlations revealed significant relations between independent observers’ ratings of adolescent social anxiety and scores reflecting the *trait* (*r* = 0.51; *p* < 0.001) and *context* (*r* = 0.24; *p* < 0.01) Satellite Model components, but not the *perspective* component (*r* = 0.02; *p* = 0.81). Similarly, bivariate correlations revealed significant relations between independent observers’ ratings of adolescent social skills and scores reflecting the *trait* (*r* = −0.48; *p* < 0.001) and *context* (*r* = −0.28; *p* < 0.001) Satellite Model components, but not the *perspective* component (*r* = −0.11; *p* = 0.21). These findings informed our tests of the unique relations of scores reflecting Satellite Model components and independent observers’ ratings of adolescent social anxiety and social skills within the Unfamiliar Peer Paradigm.

##### Unique relations

As a follow-up to our tests of bivariate relations, we constructed hierarchical regression models to test the unique effects of scores reflecting the *trait* and *context* Satellite Model components, using the analytic plan described previously. In Step 1 of the regression model testing unique effects in relation to independent observers’ ratings of adolescent social anxiety (β = 0.51; Δ*R*^2^ = 0.26; *p* < 0.001), and social skills (β = −0.48; Δ*R*^2^ = 0.23; *p* < 0.001), there was a significant effect of scores reflecting the *trait* Satellite Model component. Over-and-above effects observed in Step 1, scores reflecting the *context* component incrementally contributed a significant and moderate-magnitude effect in Step 2 for both social anxiety (β = 0.24; Δ*R*^2^ = 0.06; *p* < 0.001) and social skills (β = −0.29; Δ*R*^2^ = 0.08; *p* < 0.001). In Step 2, scores reflecting the *trait* component continued to demonstrate a significant, unique effect for both social anxiety (β = 0.51; *p* < 0.001) and social skills (β = −0.48; *p* < 0.001). Thus, in both of these regression models, scores reflecting the *trait* and *context* components demonstrated unique relations with trained independent observers’ ratings about adolescents’ social anxiety and social skills.

#### Construct validity of scores reflecting the context and perspective components

##### Bivariate relations

Bivariate correlations revealed significant relations between parent reports of adolescent-parent conflict and scores reflecting the *context* (*r* = −0.27; *p* < 0.01) and *perspective* (*r* = 0.25; *p* < 0.01) Satellite Model components, but not the *trait* component (*r* = 0.13; *p* = 0.13). These findings informed our tests of the unique relations of scores reflecting Satellite Model components and parent reports of adolescent-parent conflict in the home.

##### Unique relations

As a follow-up to our tests of bivariate relations, we constructed a hierarchical regression model to test the unique effects of scores reflecting the *context* and *perspective* Satellite Model components, using the analytic plan described previously. In Step 1 of the regression model testing unique effects in relation to parent reports of adolescent-parent conflict, there was a significant effect of scores reflecting the *context* Satellite Model component (β = −0.27; Δ*R*^2^ = 0.07; *p* < 0.01). Over-and-above effects observed in Step 1, scores reflecting the *perspective* component incrementally contributed a significant and moderate-magnitude effect in Step 2 (β = 0.23; Δ*R*^2^ = 0.05; *p* < 0.01). In Step 2, scores reflecting the *context* component continued to demonstrate a significant, unique effect (β = −0.24; *p* < 0.01). Thus, scores reflecting the *context* and *perspective* components demonstrated unique relations with parent reports of adolescent-parent conflict in the home.

#### Construct validity of scores reflecting the trait and perspective components

##### Bivariate relations

Bivariate correlations revealed significant relations between adolescent self-reports of resting arousal and scores reflecting the *trait* (*r* = 0.38; *p* < 0.001) and *perspective* (*r* = −0.27; *p* < 0.001) Satellite Model components, but not the *context* component (*r* = 0.02; *p* = 0.83). These findings informed our tests of the unique relations of scores reflecting Satellite Model components and adolescent self-reports of resting arousal.

##### Unique relations

As a follow-up to our tests of bivariate relations, we constructed a hierarchical regression model to test the unique effects of scores reflecting the *trait* and *perspective* Satellite Model components, using the analytic plan described previously. In Step 1 of the regression model testing unique effects in relation to adolescent self-reports of resting arousal, there was a significant effect of scores reflecting the *trait* Satellite Model component (β = 0.38; Δ*R*^2^ = 0.14; *p* < 0.001). Over-and-above effects observed in Step 1, scores reflecting the *perspective* component incrementally contributed a significant and moderate-magnitude effect in Step 2 (β = −0.26; Δ*R*^2^ = 0.07; *p* < 0.001). In Step 2, scores reflecting the *trait* component continued to demonstrate a significant, unique effect (β = 0.37; *p* < 0.001). Thus, scores reflecting the *trait* and *perspective* components demonstrated unique relations with adolescent self-reports of resting arousal.

## Discussion

### Main findings

Recent tests of the Kraemer et al. (2003) Satellite Model support the criterion-related validity of the approach (Makol et al., 2020). Yet, we know little about the degree to which scores reflecting the Satellite Model components (i.e., *trait*, *context*, and *perspective*) measure what they were intended to measure. Our construct validation test of the Satellite Model yielded two key sets of findings. First, as in prior work (Achenbach et al., 1987; De Los Reyes et al., 2015), we observed low-to-moderate levels of correspondence, with informants observing behavior in different contexts (i.e., parents and UUOs) displaying particularly low levels of correspondence (Table 2). In line with recent work (e.g., De Los Reyes et al., 2016; Lerner et al., 2017; Makol et al., 2019, 2021), we also found that the patterns of reports within a given set of informants (e.g., parent > teacher; youth < parent) tended to operate similarly across rated domains. In fact, when we applied the Satellite Model to integrating reports taken from adolescents, parents, and UUOs, the eigenvalues and component loadings were remarkably similar across the domains measured with these reports (i.e., social anxiety, avoidance behaviors, fears of negative evaluation, depression, and impairments; see Table 3). These findings culminated in creating multi-item scales of the *trait*, *context*, and *perspective* components derived from the Satellite Model; each scale displayed high internal consistency (i.e., α > 0.80; see Nunnally and Bernstein, 1994), particularly for short, six-item scales (see also Youngstrom et al., 2019).

Second, we curated a set of criterion variables that, like the Satellite Model components themselves, systematically varied from each other in the contexts, perspectives, and sources of measurement used to create them. The multi-item *trait*, *context*, and *perspective* scales yielded distinct patterns of relations with this diverse set of criterion variables (Table 4). Further, these patterns of relations aligned with prior work on how adolescent, parent, and UUO reports relate to each other *and* these criterion variables. For instance, prior work indicates that all three of these informants’ reports individually relate to trained independent observers’ ratings of adolescent behavior (Glenn et al., 2019; Botkin et al., 2021; Rezeppa et al., 2021). A logical extension of this observation is that scores reflecting the *trait* component would relate to independent observers’ ratings, which they did. However, recall that these independent observers based their ratings on adolescents’ behavior within interactions that occur in non-home contexts (i.e., within the Unfamiliar Peer Paradigm). Thus, we observed a “match” between the context used to estimate observed adolescent social anxiety and social skills and the *context* component of the Satellite Model. Further, trained independent observers made their ratings from a perspective that, by construction, is distinct from the adolescents, parents, and UUOs whose reports were integrated using the Satellite Model. This explains why we observed non-significant relations between independent observers’ ratings and the *perspective* component.

We observed similarly coherent patterns with our two other criterion variables. Specifically, parent reports of home-specific psychosocial experiences (adolescent-parent conflict) displayed unique relations with scores reflecting the *context* and *perspective* components, but not the *trait* component. In light of the low correlations between parent and UUO reports (Table 2), it makes sense that a criterion variable based on the parent report would fail to display relations with scores reflecting the *trait* component, given that, by design, the trait component loads strongly onto all of the informants’ reports (Table 3). Thus, we observed a “match” between the context and perspective used to estimate adolescent-parent conflict and the *context* and *perspective* components of the Satellite Model.

In contrast to the effects observed with parent reports of adolescent-parent conflict, adolescent self-reports of a context-neutral, internal experience (resting arousal) displayed unique relations with scores reflecting the *trait* and *perspective* components, but not the *context* component. In line with the Satellite Model, the relation between the *perspective* component and adolescent resting arousal stems from the fact that we estimated resting arousal in a way that “matched” a self-perspective (i.e., using adolescent self-reports). Yet, how do we explain the *trait* component relation? Here too, we can look to prior work. Specifically, resting arousal relates to informants’ reports of youth anxiety (see Monk et al., 2001; Thomas et al., 2012a). Further, recent work finds that adolescents’ self-reported arousal relates to not only adolescent self-reports on mental health surveys but also the survey reports of other informants like peer confederates and UUOs (Rausch et al., 2017; Karp et al., 2018; Qasmieh et al., 2018; Rezeppa et al., 2021). Thus, multiple informants in our Satellite Model—namely adolescents and UUOs—made survey reports about adolescent mental health that each relate to adolescent self-reports of resting arousal. Further, informants like adolescents and UUOs vary in the contexts in which they observe behavior. This logically results in detecting links between self-reported arousal and scores reflecting the *trait* component. Because no coherent pattern exists between the context-specificity of these informants, we would not expect relations between scores reflecting the *context* component and a context-neutral criterion variable such as resting arousal. Taken together, our findings support the ability to create multi-item, internally consistent scales reflecting components derived from the Satellite Model, and in a way that results in domain-relevant scores, essentially data pertaining to what these components were designed to reflect.

### Research and theoretical implications

Our study has important implications for research and theory germane to youth mental health assessments. In particular, researchers in youth mental health have both long-observed informant discrepancies in assessments of youth mental health (e.g., Lapouse and Monk, 1958; De Los Reyes, 2013), and *theorized* that these discrepancies might be reflective of the situational specificity of youth mental health concerns (Achenbach et al., 1987). We highlighted a series of studies over the last decade that support the notion of situational specificity (for reviews, see De Los Reyes et al., 2019a,b), as well as a conceptual model to guide work on these issues (OTM; De Los Reyes et al., 2013). Yet, there is uncertainty about how to *integrate* multi-informant data in a way that preserves both the *unique variance* that each report contributes, as well as the *common variance* these reports collectively contribute.

Essentially, the developers of the Satellite Model sought to instantiate notions of situational specificity within integrated scores taken from multi-informant assessments. When leveraged to integrate multi-informant assessments of adolescent mental health, our study supports these notions about what scores taken from the model reflect. In particular, the model appears to capture domain-relevant common variance (i.e., *trait*) as well as domain-relevant unique variance (i.e., *context* and *perspective*). This balance between emphasizing both common variance and domain-relevant unique variance is a rarity in integrative approaches. In fact, available approaches largely emphasize common variance and treat unique variance as measurement confounds (see Offord et al., 1996; Rubio-Stipec et al., 2003; Youngstrom et al., 2003; Eid et al., 2008; Valo and Tannock, 2010; Watts et al., 2021). In these respects, we see the Satellite Model as facilitating the alignment of conceptual and measurement models of youth mental health assessment. At the same time, we suspect it is not the only model that can facilitate this alignment. Thus, a key direction for future research will involve refining existing approaches to similarly balance the measurement of common variance and domain-relevant unique variance, as well as design new approaches that instill such balance when integrating multi-informant data.

### Clinical implications

Our findings also have important clinical implications. Indeed, as an approach to data integration, the PCA procedures used to estimate the components of the Satellite Model result in sample-level estimates of component loadings, but also individual-level scores for each participant in the sample. This comprises the first step in testing whether scores designed to estimate these components reflect the domains they were intended to reflect (i.e., *trait*, *context*, and *perspective*). This is also the first step in developing approaches that facilitate interpreting multi-informant data in service settings with individual clients. In these respects, recent work charts a path toward developing these individual-level approaches (for a review, see Talbott and De Los Reyes, 2022). As others have noted (e.g., Makol et al., 2020), PCA is like any other data aggregation technique. In fact, the way in which PCA is incorporated into the Satellite Model shares important similarities with how others have applied this and related factor analytic procedures to enhance the interpretability of individual-level summary scores from client assessments (e.g., total scores from a parent report on a behavioral checklist; see Achenbach and Rescorla, 2001). Consequently, we see two key directions for future research.

First, scaling up the Satellite Model will involve developing normative scores to reflect its constituent components. This would require not only large, representative samples to produce model estimates, but also new assessment strategies for collecting reports from informants that do not tend to appear in traditional clinical assessments of youth mental health, namely UUOs. As we have articulated previously (Rezeppa et al., 2021), the practical value of our approach to collecting reports from UUOs lies in our reliance on (a) untrained raters and (b) videotaped segments of therapeutic activities already widely implemented in evidence-based interventions administered to youth clients (i.e., therapeutic exposures; Cannon et al., 2020). As such, we designed our application of the Satellite Model in a way that optimizes its clinical feasibility. In this respect, we encourage future research that probes the clinical feasibility of this approach.

The second step will involve developing validation approaches to interpret scores taken from the Satellite Model with individual cases. That is, to simply assume that these scores accurately reflect the domains they were designed to reflect when working with individual clients would be akin to engaging in the naming fallacy described previously with respect to how researchers interpret the results of factor analytic procedures (see Kline, 2016). Yet, this too is an issue that assessors might overcome with measurement at the individual case level. For instance, consider an assessor who has access to both the multi-informant data used to develop normed Satellite Model scores *and* clients’ scores on criterion measures that vary on the context, perspective, and source of measurement. With these data, an assessor can create a client-level version of our study. The goal would be to verify that scores reflecting the Satellite Model components are operating as intended and that patterns of scores reflecting the *trait*, *context*, and *perspective* components “match” or demonstrate consistencies with the battery of criterion variables (see Table 4). These issues merit further study.

### Limitations

The limitations of our study highlight directions for future research. A key aim of this study involved testing links between multi-informant assessments of adolescent mental health that incorporated reports from adolescents, parents, and UUOs. Importantly, the UUOs based their reports on observations of adolescents interacting with unfamiliar peer confederates within the Unfamiliar Peer Paradigm. These confederates were undergraduate and post-baccalaureate personnel who we trained to simulate unfamiliar, same-age peers. Consistent with prior work (Deros et al., 2018; Cannon et al., 2020), we only leveraged the assistance of personnel who appeared youthful and could reasonably appear to adolescents as same-age, unfamiliar peers (e.g., wearing age-appropriate casual clothing, no facial hair for male confederates). Yet, peer confederates were a different age relative to our study participants. Further, we did not examine the degree to which adolescents believed that these confederates were their own age. Importantly, in prior work, we learned that adolescents’ reactions to unfamiliar peer confederates within this paradigm predict their reactions to a well-established task where they are (a) told explicitly that they would be interacting with same-age, unfamiliar peers; and (b) provided with photographic stimuli to support this element of the task (i.e., Cyberball; see Karp et al., 2018). Nevertheless, we cannot be certain that adolescents’ reactions to the Unfamiliar Peer Paradigm would have been identical to their reactions to interactions with same-age, unfamiliar peers in general. Further, we recruited participants within a fairly limited age range of 14–15 year olds. Thus, our findings might not generalize to adolescents within earlier and later developmental periods as well as from different geographical locations. Future research should examine the generalizability of the findings when using age-matched adolescents as peer confederates, and within samples of older and younger adolescents.

## Concluding comments

Despite the ubiquity of informant discrepancies in multi-informant assessments of youth mental health, no consensus guidelines exist for integrating informants’ reports. Without clarity in these areas, researchers and clinicians lack guidance on how to leverage multiple informants’ reports to make clinical decisions. In this paper, we described approaches to integrating multi-informant data, with a particular emphasis on the Kraemer et al. (2003) Satellite Model, a promising approach designed to yield accurate estimates of both common variance and domain-relevant unique variance observed in multi-informant data. Along these lines, we performed a thorough construct validation test of the Satellite Model, which included (a) estimating the levels of correspondence among multiple informant reports, (b) applying the Satellite Model to a battery of multi-informant surveys on parallel instruments, (c) testing the internal consistencies of aggregated “total scores” of Satellite Model common and unique variance components from this battery, and (d) testing the construct validity of these total scores using a multi-modal battery of domain-relevant criterion variables. Our findings suggest that the Satellite Model instantiates in measurement the Achenbach et al. (1987) notion of situational specificity. That is, the Satellite Model captures in measurement the notion that informant discrepancies manifest because: (a) youth vary as to where they display mental health concerns and (b) the informants from whom assessors solicit reports (e.g., parents, teachers, youth, and their peers) vary in the contexts and perspectives from which they observe the youth. Taken together, the Satellite Model facilitates unifying conceptual and measurement models of youth mental health. As such, a key direction for future research involves testing versions of the Satellite Model that can be applied to individual or case-level data.

## Data availability statement

The raw data supporting the conclusion of this article will be made available by the authors, without undue reservation.

## Ethics statement

The studies involving human participants were reviewed and approved by University of Maryland College Park (UMCP) IRB. Written informed consent to participate in this study was provided by the participants’ legal guardian/next of kin.

## Author contributions

NC wrote the first draft of the manuscript and edited the manuscript. SR and MW contributed to the data analysis and edited the manuscript. AD conceptualized the problem, designed the study, acquired study funding, organized and executed the data analyses, edited the manuscript, and supervised NC in writing the first draft of the manuscript. All authors approved the manuscript and agreed to be accountable for all aspects of the work.
