# Supplementary material for: Integrating multi-informant reports of youth mental health: A construct validation test of Kraemer and colleagues’ (2003) Satellite Model
Source: Front Psychol. 2022 Jul 28;13:911629. doi: 10.3389/fpsyg.2022.911629 (PMC9371006; doi:10.3389/fpsyg.2022.911629)
Supplement: Supplementary file 1 [file Data_Sheet_1.PDF]

Integrating Multi-Informant Reports of Youth Mental Health: A Construct Validation Test of Kraemer and Colleagues' (2003) Satellite Model

Natalie Charamut[1], Sarah J. Racz[1], Mo Wang[2], Andres De Los Reyes[1*]

[1]Comprehensive Assessment and Intervention Program, University of Maryland at College Park, Department of Psychology, College Park, MD, USA
[2]Department of Management, University of Florida, Gainesville, FL, USA

* Correspondence:
Andres De Los Reyes (adlr@umd.edu)

ONLINE SUPPLEMENTARY MATERIAL

Integrating Multi-Informant Reports of Youth Mental Health: A Construct Validation Test of Kraemer and Colleagues' (2003) Satellite Model

ONLINE SUPPLEMENTARY MATERIAL

Characteristics of Unfamiliar Untrained Observers (UUOs)

Demographics. Two sets of UUOs provided reports on adolescents in this sample. One set of UUOs made reports of adolescents specifically on the SAFE. These 28 "SAFE-only" UUOs had a mean age of 21.07 years (*SD* = 2.92 years) and included 21 females and 7 males. The SAFE-only UUOs self-identified their racial/ethnic background as African American or Black (10.7%); Asian American or Asian (25%); Hispanic or Latino/a (Spanish) (17.9%); or White, Caucasian American, or European (53.6%) (rates total above 100% because UUOs could select multiple response options). The SAFE-only UUOs self-reported their current educational status at the time they completed their reports as an undergraduate sophomore (25%), junior (32.1%), senior (25%); a post-baccalaureate trainee (i.e., not yet matriculated in a graduate program; 7.1%); or a master's level graduate student (10.7%).

A second set of UUOs made reports of adolescents on an additional five survey measures to complete the six-survey battery (i.e., SIAS, SPS, BFNE, WSASY, & BDI-II). These 45 UUOs who provided reports about adolescents on these five measures had a mean age of 19.96 years (*SD* = 1.61 years) and included 36 females and 9 males. The UUOs self-identified their racial/ethnic background as African American or Black (11.1%); Asian American or Asian (33.3%); Hispanic or Latino/a (Spanish) (15.6%); White, Caucasian American, or European (48.9%); American Indian or Native American (2.2%); or "other" (i.e., Middle Eastern, Iranian; 4.4%) (rates total above 100% because UUOs could select multiple response options). The 45 UUOs self-reported their current educational status at the time they completed their reports as an undergraduate freshman (13.3%), sophomore (40%), junior (17.8%), senior (15.6%); a post-baccalaureate trainee (i.e., not yet matriculated in a graduate program; 4.4%); or a master's level graduate student (8.9%).

Procedures for gathering UUO reports. Based on archival videos of the 134 adolescents' participation in the Unfamiliar Peer Paradigm, UUOs were randomly assigned to each observe the video recordings of up to five adolescents. For each adolescent they observed, UUOs viewed recordings of their social interactions during the Unfamiliar Peer Paradigm, and completed their survey reports about the adolescent immediately following viewing these recordings. We masked the UUOs to all clinical characteristics of the adolescents about whom they provided reports, including referral status and all clinic data (e.g., scores on other instruments). Further, UUOs did not participate as peer confederates with the adolescents about whom they provided reports. In this respect and similar to our procedures for peer confederates, UUOs had no prior contact with the adolescents about whom they provided reports. In essence, we selected UUOs and assigned them to videos of adolescents, such that we could ensure their unfamiliarity with these adolescents.

Characteristics and Training of Independent Observers

For each adolescent, two trained independent observers viewed archived videotapes of their participation in the Unfamiliar Peer Paradigm. All trained independent observers received

training on how to use the behavioral ratings of adolescent social skills. Trained independent observers consisted of post-baccalaureate and undergraduate research assistants. We masked trained independent observers to adolescents' referral status and they did not have access to adolescents' clinical information. Further and as with the UUOs, none of the trained independent observers participated as a peer confederate in the Unfamiliar Peer Paradigm.

To train independent observers on the coding scheme described below, a team of eight to ten researchers (i.e., undergraduate students, post-baccalaureate research assistants, graduate students, and faculty) participated in consensus coding meetings in which team members simultaneously viewed videos of all the social interaction tasks (i.e., SSIT, UCT, IST) performed by five adolescent participants in the sample. Following each task viewing, team members independently rated the adolescent in the video on the levels of social skills they displayed during the task, using the coding scheme below (i.e., a rating for each of the five SSIT role-plays, a rating for UCT, a rating for IST). After each team member made their ratings for a task, the entire team discussed the ratings. During this discussion, the team resolved discrepancies among ratings, and came to a final consensus rating for social skills displayed by the adolescent participant performing the task. We repeated this process for each of the five participants across all seven tasks (i.e., seven social skills consensus ratings per participant).

After creating the consensus ratings for five training cases, we trained the independent observers described previously. Each trained independent observer independently viewed videos for the five training cases and made seven social skills ratings per case. After making their training ratings, we calculated intraclass correlation (ICC) statistics to assess inter-rater reliability between each trained independent observer and the consensus ratings. We set a threshold of a mean ICC of .80 to determine whether a trained independent observer successfully passed the training stage. All trained independent observers passed our criterion ICC, and following training, these observers coded the cases in the sample to which they were assigned.
